# Commensal Streptococcal Infective Endocarditis of the Native Mitral Valve in a Transcatheter Aortic Valve Replacement (TAVR) Patient: A Heartful, a Handful, and a Mouthful

**DOI:** 10.7759/cureus.42565

**Published:** 2023-07-27

**Authors:** Michael Kozak, Mark Kozak

**Affiliations:** 1 Internal Medicine, Penn State College of Medicine, Hershey, USA; 2 Cardiovascular Medicine, Penn State College of Medicine, Hershey, USA

**Keywords:** aortic valve, tavr, oral flora, anaerobe, ie, infective endocarditis

## Abstract

An 88-year-old woman with an extensive medical history presented to the hospital with altered mental status, vague abdominal pain, and dysuria. A previous transcatheter aortic valve replacement (TAVR) prosthesis was known to be failing and was suspected to have acquired a vegetation. No other infective endocarditis (IE) stigmata were present. Fortunately, the work-up for replacement was allowed to proceed with a broader cardiac examination from which a mitral vegetation was identified and IE then treated.

## Introduction

The incidence of infective endocarditis (IE) is 34-36 cases per million person-years with a peak incidence between the ages of 70 and 80 years. With a marked male predominance of almost 3:1 that decreases with age [1-2], almost one-third of all cases occur in patients with implanted equipment [3]. *Staphylococcus aureus* is now the most common causative organism [4]; other frequently encountered organisms causing IE include *Streptococcus gallolyticus*, *Viridans streptococci*, coagulase-negative staphylococci, HACEK (*Haemophilus* species, *Aggregatibacter actinomycetemcomitans*, *Cardiobacterium hominis*, *Eikenella corrodens*, *Kingella kingae*) organisms, and enterococci [5]. Entry is most often cutaneous, oral, or digestive. Despite advances in treatment, the prognosis for IE remains poor; acute mortality is nearly 25% and has not decreased much over time [3,6]. This is certainly not the first description of IE caused by *Streptococcus gordonii* [7-9]; however, in the patient with underlying cardiac pathology and transcatheter aortic valve replacement (TAVR) bioprostheses, an understanding of the approach and considerations for prompt and accurate diagnosis is essential as TAVR becomes more frequently employed in increasingly younger patients [10].

## Case presentation

An 88-year-old woman had a history of TAVR, a percutaneous coronary intervention of the left circumflex artery, transient ischemic attack, hypertension, congestive heart failure, type II diabetes mellitus, and pulmonary artery hypertension presented with 24 hours of drowsiness and mental status change, abdominal pain, and dysuria.

Vital signs on arrival were notable for hypotension at 102/40 and tachypnea to 26 respirations per minute requiring 4 L oxygen to bring saturation above 98%. Glasgow's coma scale score was 13 on arrival. Laboratory tests showed anemia (hemoglobin of 9.5), leukocytosis (only mild to 21,700), and acute kidney injury. Cardiac examination was significant for a mid-systolic blowing murmur at the left upper sternal border and apex. An ECG was unchanged from the previous one. CT brain and chest X-rays were unremarkable. We started empiric antibiotics, and blood cultures grew *Streptococcus gordonii*, a common oral viridans strep.

**Figure 1:**
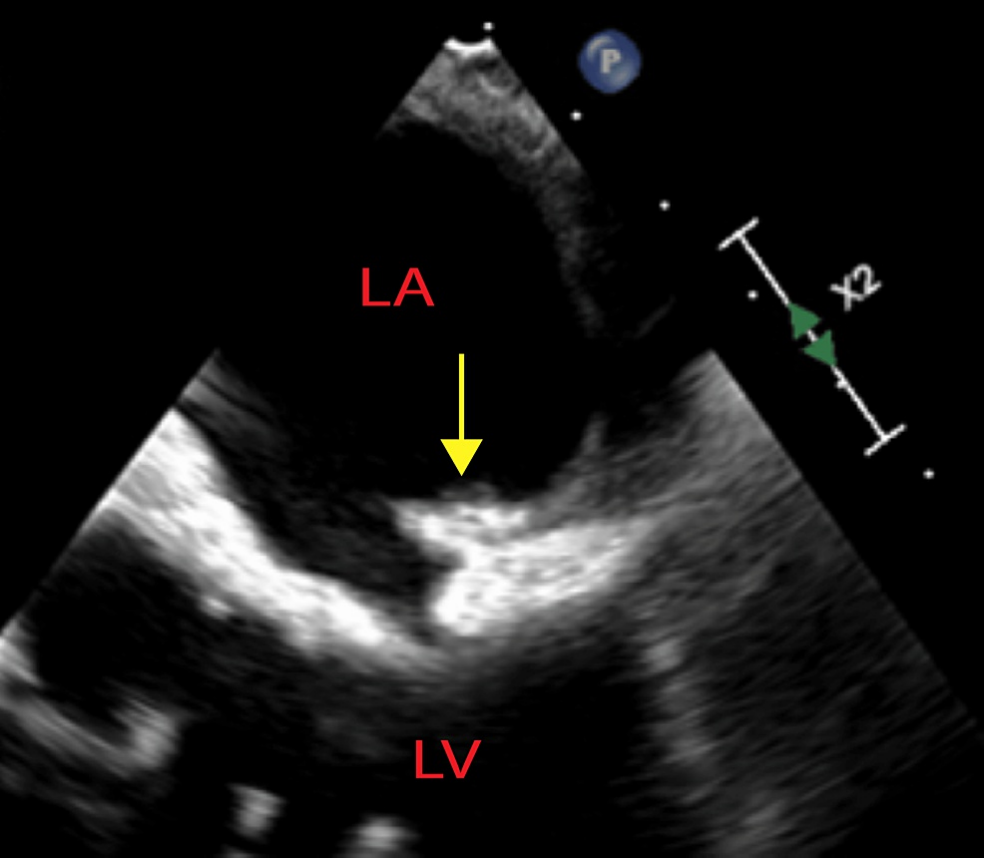
Mid-esophageal four-chamber TEE. LV and LA were identified. Vegetation identified with a yellow arrow LV: left ventricle, LA: left atrium

**Figure 2:**
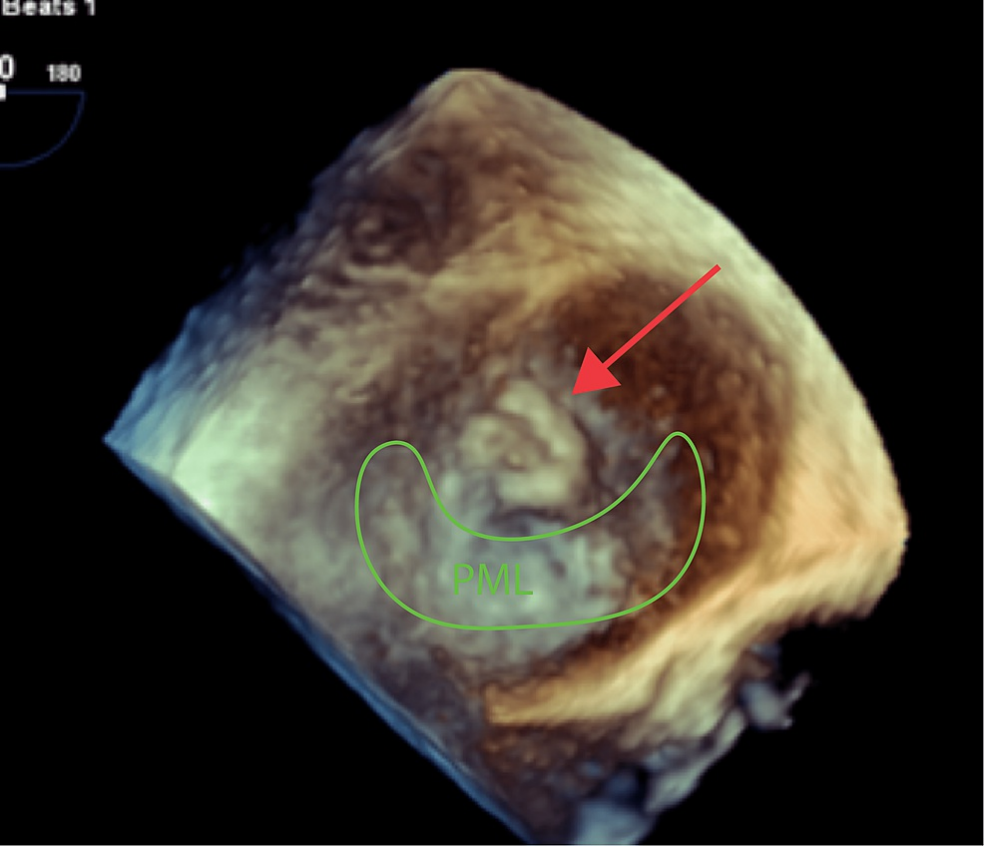
3D reconstruction from TEE showing mitral valve vegetation on the atrial side of the leaflet (red arrow) PML: posterior mitral leaflet

**Figure 3:**
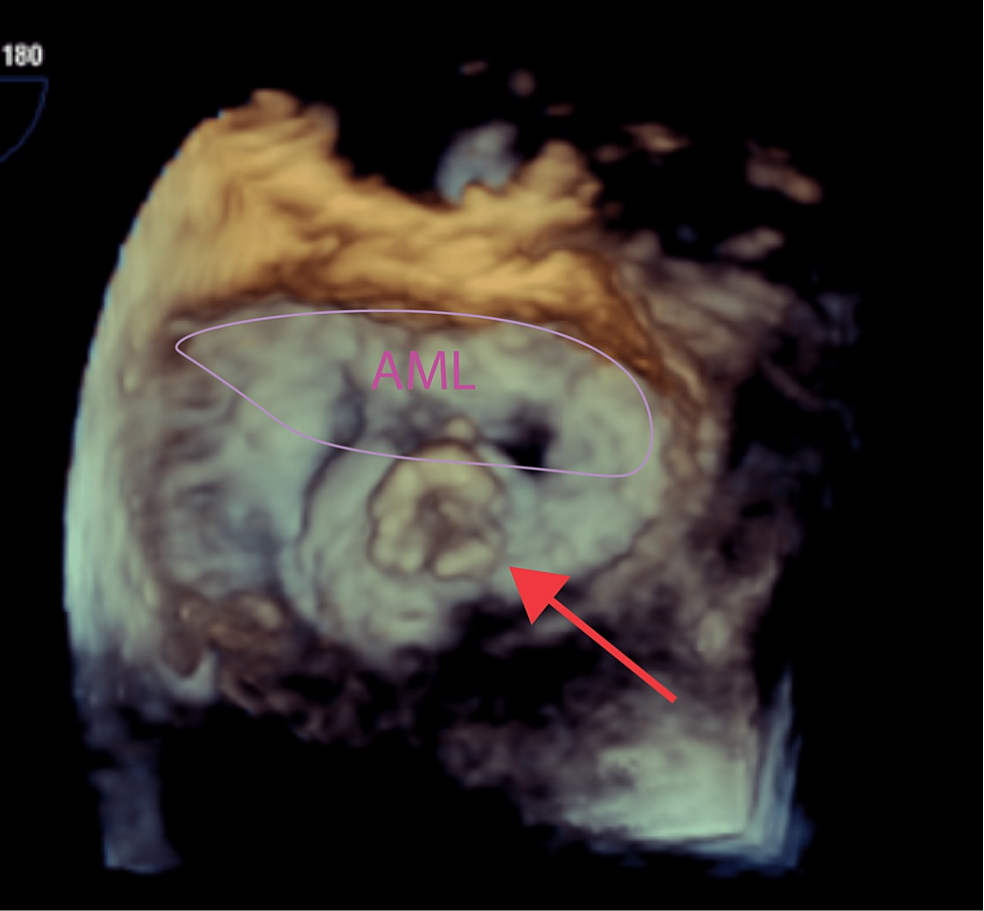
3D reconstruction from TEE showing mitral valve vegetation on the atrial side of the leaflet (red arrow). Anterior mitral leaflet approximately outlined in purple AML: anterior mitral leaflet

The patient had an existing appointment for a transesophageal echocardiogram (TEE) for TAVR evaluation in the setting of suspected recurrent aortic stenosis on the third day after admission. The patient’s condition improved only modestly with antibiotics (ceftriaxone brought the white count down to 15k), with no change in mental status. Her blood pressure improved with fluids to 130/50. Her oxygen requirement was unchanged. The TEE on day three demonstrated a vegetation on the mitral valve in the atria, toward the free end of the anterior mitral leaflet (Figures 1-3), not the bioprosthetic originally suspected. She met both major criteria, a visualized vegetation by echocardiography and a positive blood culture of the Duke criteria [8]. A panoramic dental X-ray then identified a molar abscess; she underwent a tooth extraction in the hospital, a central line was placed, and she was started on six-week IV antibiotics (ceftriaxone).

## Discussion

Normally the endocardium is resistant to bacterial seeding. The development of IE requires an injury to the endocardium concomitant with bacterial exposure. Typical endocardial insults include diseased valves, particulate matter, turbulent flow stress, and catheter passage as occurs during TAVR. The damaged endocardium first accumulates platelets permitting colonization by bacteria from the bloodstream [11]. Bacteria then proliferate, sometimes forming protective biofilms, with subsequent shedding into the bloodstream, resulting in many of the hallmark clinical findings.

The presentation of left-sided IE often begins with constitutional symptoms. A new murmur or a change in an old murmur is highly suggestive. Many of the presenting symptoms of IE, both septic and immune, are related to emboli dislodging from the vegetation; hallmark findings include Roth spots, Osler's nodes, Janeway lesions, splinter hemorrhages, splenic infarctions, cerebral emboli, petechiae, and clubbing [12]. The presentation of right-sided IE is somewhat different and is not discussed [13].

IE is formally diagnosed using the Duke criteria [12] where signs and symptoms are divided into major and minor criteria. Diagnosis requires two major criteria, one major and three minor, or five minor criteria.

The incidence of endocarditis after TAVR is approximately 0.87% per year, with a declining trend over time [14] which is almost identical to the risk after surgical aortic valve repair [15-16]. In TAVR patients, the echocardiographic diagnosis of post-TAVR endocarditis is more challenging owing to the method of the valve implantation, the absence of a decalcification area, and the remnant of the native calcified aortic valve which has been pressed to the periphery of the original annulus. Additionally, vegetation and abscess are very difficult to identify by TEE or TTE in the free space between the transcatheter and native aortic valve. The transcatheter valves consist of metal which creates a shadowing effect further complicating echocardiographic diagnostics, especially for smaller vegetations [17-19]. Echocardiographic images were considered normal or inconclusive in 47.7% of echocardiographic studies. The modified Duke criteria are quite accurate in TAVR IE, in a large study definite in 63.1% and possible in 36.9% of IE patients. Additionally, nearly all TAVR IE patients were blood-culture positive [10].

Satellite endocarditis of the mitral valve as a result of the direct contact of the vegetated aortic transcatheter valve with the mitral apparatus has been demonstrated in 24% of prosthetic valve endocarditis cases [20], while secondary mitral valve involvement is estimated in 10% of patients with native aortic IE in the "mitral kissing vegetation," which refers to large aortic vegetations prolapsing into the left ventricular outflow tract and making contact with the ventricular aspect of the anterior mitral leaflet causing secondary infection [21].

However, both aforementioned studies begin with prosthetic aortic valve endocarditis. Evidence for any increased risk of isolated mitral valve endocarditis in the TAVR patient either does not exist or has not yet been published, suggesting that the native mitral valve in a TAVR patient is no more or less likely to be the site of vegetation in a patient with IE than in the general population.

The effect of TAVR, in general, on the mitral apparatus is not wholly predictable given the variability in anatomy and deployment. The mitral valve may be subject to changes in both geometry and structure during TAVR that can potentially cause functional mitral regurgitation leading to potential endocardial injury or accentuating the problems of a pathological mitral valve [22].

Several patient and procedural factors may predispose to mitral apparatus disruption and/or downstream injury predisposing to bacterial seeding and vegetation formation. Some of the more common factors that predispose to mitral disruption include under-sizing of the TAVR valve, under-expansion of an appropriately sized TAVR valve due to aortic root calcification, low implantation of the TAVR valve, insufficient TAVR valve anchoring, the presence of a surgical mitral bioprosthetic valve, initial unstable TAVR valve position, and basal septal bulging [20].

For the patient presenting with a pathognomonic sign or symptom, few clinicians will miss the diagnosis. Osler’s nodes, Janeway lesions, and splinter hemorrhages are firmly fixed in the minds of all physicians. However, these "classic" signs are relatively uncommon with data suggesting that these three are present in less than 10% of patients with IE; additionally, TTE and TEE both have significant limitations for diagnosis in TAVR patients when the aortic valve or prosthetic are affected [17-19].

In our case, our patient would only have met one major and two minor Duke criteria (which would have been “possible IE” by the modified Duke criteria [23]) prior to our finding the vegetation on the mitral valve which was itself fortuitous because the patient had a TEE scheduled for an unrelated reason and, thus, was performed near the time when the first concern for IE arose. Additionally, her history of TAVR likely adds no increased risk of primary mitral valve IE, while TAVR only increases the annual risk of IE by a factor of 2-3 [1-2,14]. In fact, the case presented here, in hindsight, likely warranted a further investigation of her native and bioprosthetic aortic valves due to the relatively low incidence of primary mitral valve endocarditis in patients with bioprostheses and the relatively common occurrence of secondary mitral involvement by "mitral kissing" which occurs with TAVR IE [20-21].

## Conclusions

Patients with prosthetic valves, including bioprostheses, are at an increased risk for IE, but imaging work-up for these patients should remain comprehensive when IE is suspected and not immediately confirmed on the interrogation of the prosthesis. IE is on the differential of all providers approaching bacteremia; however, the Duke criteria are merely one tool, with good sensitivity and modest specificity. Imaging is essential to identify vegetation which is the upstream cause of most of the hallmark signs of IE, but echocardiographic imaging has substantial limitations in TAVR patients. Empiric antibiotics are started immediately when IE is suspected. Concurrently, robust history and additional testing should be performed to confirm the source of IE and appropriate interventions performed, in our case that was a tooth extraction with washout.
